# Knowledge is power? Cervical cancer prevention in female OB/GYNs compared to other female physicians

**DOI:** 10.3389/fpubh.2023.1269393

**Published:** 2023-09-15

**Authors:** Gal Hershkovitz, Yifat Ochshorn, Nadav Michaan, Elisheva Fiszer, Dan Grisaru, Yael Raz

**Affiliations:** ^1^Department of Obstetrics and Gynecology, Lis Maternity Hospital, Tel Aviv Sourasky Medical Center, Tel Aviv, Israel; ^2^Sackler Faculty of Medicine, Tel Aviv University, Tel Aviv, Israel; ^3^Division of Anesthesiology, Pain and Intensive Care, Tel Aviv Sourasky Medical Center, Tel Aviv, Israel

**Keywords:** cervical cancer, cervical screening, HPV vaccine, female physicians, OB/GYN, prevention

## Abstract

Cervical cancer (CC) screening and prevention are crucial responsibilities of obstetrician-gynecologists (OB/GYNs). Our study aimed to investigate whether knowledge impacts OB/GYNs’ (*n* = 42) adherence to CC prevention measures by comparing them to non-OB/GYN physicians (*n* = 80). An anonymous questionnaire collected demographic information, personal screening habits and evaluated their knowledge of CC prevention. Results revealed that OB/GYNs exhibited superior knowledge of CC risk factors and prevention compared to non-OB/GYNs. Of note, a lower percentage of OB/GYN residents correctly identified the recommended upper age limit for cervical screening and for HPV vaccination compared to attending OB/GYNs (50% vs. 83%, *p* = 0.04 and 11% vs. 50%, *p* = 0.01, respectively). Despite these findings, most physicians from both groups recommended HPV vaccination. Cervical screening rates were similar between OB/GYNs and non-OB/GYNs (75% vs. 83%, *p* = 0.3). Half of OB/GYNs initiated their own cervical screening, similar to non-OB/GYNs. Interestingly, residents had higher HPV vaccination rates compared to attending physicians, irrespective of specialty (OB/GYNs – 38.89% vs. 4.76%, *p* = 0.0149; non-OB/GYNs – 51.06% vs. 15.38%, *p* = 0.0028). In conclusion, contrary to the assumption that physicians prioritize personal well-being, our study reveals the opposite. While skilled in guiding patients through CC screening and prevention, female OB/GYNs often neglect their own health. OB/GYNs must also be educated and supported in safeguarding their health, setting an essential example for patients.

## Introduction

Cervical cancer (CC) is the fourth most common cancer in women worldwide (1) and the leading cause of cancer death in women in lower-resource countries (2). Infection with Human Papillomavirus (HPV), the leading pathogen causing the disease, can be identified in 99.7% of invasive CC cases (3). Known risk factors for HPV infection and CC development include early onset of sexual activity, multiple sexual partners, prior history of sexually transmitted diseases (STD), immune suppression, smoking, low socioeconomic status, young age at first delivery (<20 years old) and high parity (>3 births) (4).

CC incidence and mortality rates in Israel are among the lowest in high-resourced countries (5), although the frequency of premalignant lesions is similar to that in other populations. Though data on HPV genotypes in Jewish Israeli CC patients is limited, the prevalence of HPV16 and HPV18, the most common high-risk types, is similar to that reported in North America and Europe (6). Most characteristics, except certain traditional habits, are also similar to those in other populations (7), leaving the low incidence of CC in Israel currently unexplained.

Cervical screening in Israel is accessible and funded. Treating physicians play a pivotal role in raising awareness and encouraging their patients to comply with cervical screening recommendations. The Israeli Society of Obstetrics and Gynecology recommends cervical screening in all women aged 25–65 years, using tri-annual Pap testing or HPV typing (8).

The first HPV vaccine was approved by the FDA in 2006. Available vaccines protect against two, four or nine HPV types and they all include vaccination against HPV-16 and HPV-18, the most prevalent high-risk types detected in CC specimens (9). The WHO recommends HPV vaccines as part of routine vaccinations in all countries, along with other preventive measures (10). While the vaccines efficacy is very high, it does not eliminate the risk of CC and screening is still required. HPV vaccination has been part of the governmental immunization program in Israel since 2013 and is routinely administered to girls and boys in 8th grade since 2016. The vaccine is further subsidized by the state for individuals up to 26 years old who were not vaccinated as part of the program and in certain indications can also be administered up to the age of 45 (11).

Several studies have questioned the intuitive correlation between knowledge and cervical screening performance among heterogeneous groups of healthcare providers. In all of these studies, the majority of respondents were nurses and midwives with only a small percentage of physicians (12–15). They all found low rates of test execution (13, 14) with the most common explanation given for the low performance rate being “forgetfulness and neglect.”

The training and knowledge of physicians differ significantly from that of other medical professionals. In addition, it is the obstetrician-gynecologist’s (OB/GYN’s) responsibility to schedule and perform cervical screening and affirm HPV vaccination. These reasons led us to focus our study on physicians alone. Interviewing female OB/GYN physicians and physicians of other specialties (“non-OB/GYNs”) we aimed to evaluate their knowledge regarding CC, assuming that it will increase OB/GYN physicians’ compliance with CC screening and prevention.

## Materials and methods

The study was approved by the institutional review board (IRB) at the Tel Aviv Sourasky Medical Center. Informed consent was waived by our IRB due to the anonymous nature of the questionnaires.

We approached OB/GYN and non-OB/GYN female physicians at all levels of seniority who worked at the Tel Aviv Sourasky Medical center during the period of February 2018 to February 2020 in request to answer an anonymous 2-part -questionnaire. Most of the printed questionnaires were delivered to the different hospital wards where they were distributed to the female physicians while a small fraction of them were delivered via electronic mail.

### Questionnaires

The first part of the questionnaire included 27 questions regarding personal data (Supplementary File S1). The second part of the questionnaire included 12 knowledge questions regarding CC risk factors, cervical screening tests and HPV vaccination (Supplementary File S2). During the study period, HPV testing was incorporated as the routine cervical screening test in one of the four HMO (health maintenance organization) groups operating in Israel and was available at the physician’s discretion in the other HMO groups. Since the screening interval and sampling method are the same, and to avoid confusion, we chose to use the inclusive term “cervical screening” in our questionnaires, referring to both screening options.

### Risk factor awareness index

As part of the knowledge assessment (Supplementary File S2, question 12) participants were asked to identify relevant risk factors for CC out of 7 options given. Each correct identification (either identifying a relevant risk factor as “TRUE” or not - identifying a false risk factor as “TRUE”) granted the questioned physician one point for a maximum of 7 points. The sum of points comprised the individual physician’s “Risk factor awareness index.”

### Statistical analysis

We anticipated a 25% difference in knowledge regarding CC screening guidelines between OB/GYNs and non-OB/GYNs. Thus, a total of 120 participants in a 1:2 ratio were recruited in order to obtain a power of 80% at a significance level of *p* < 0.05 (2-sided test). Statistical analysis was performed with the use of the SPSS statistic software (version 22; IBM ® Corporation, NY, United States) and GraphPad Prism (version 8.4.3; GraphPad software, CA, USA). Distribution of variables was evaluated using histograms and Q-Q plots. Kruskal- Wallis test with Dunn’s multiple comparisons test were used to compare non-parametric continuous variables. Fishers’ exact test was used to determine association between categorical variables. All statistical analyses were 2 sided and *p* < 0.05 was considered statistically significant. Results are presented as mean and standard deviation (SD).

## Results

**Figure 1:**
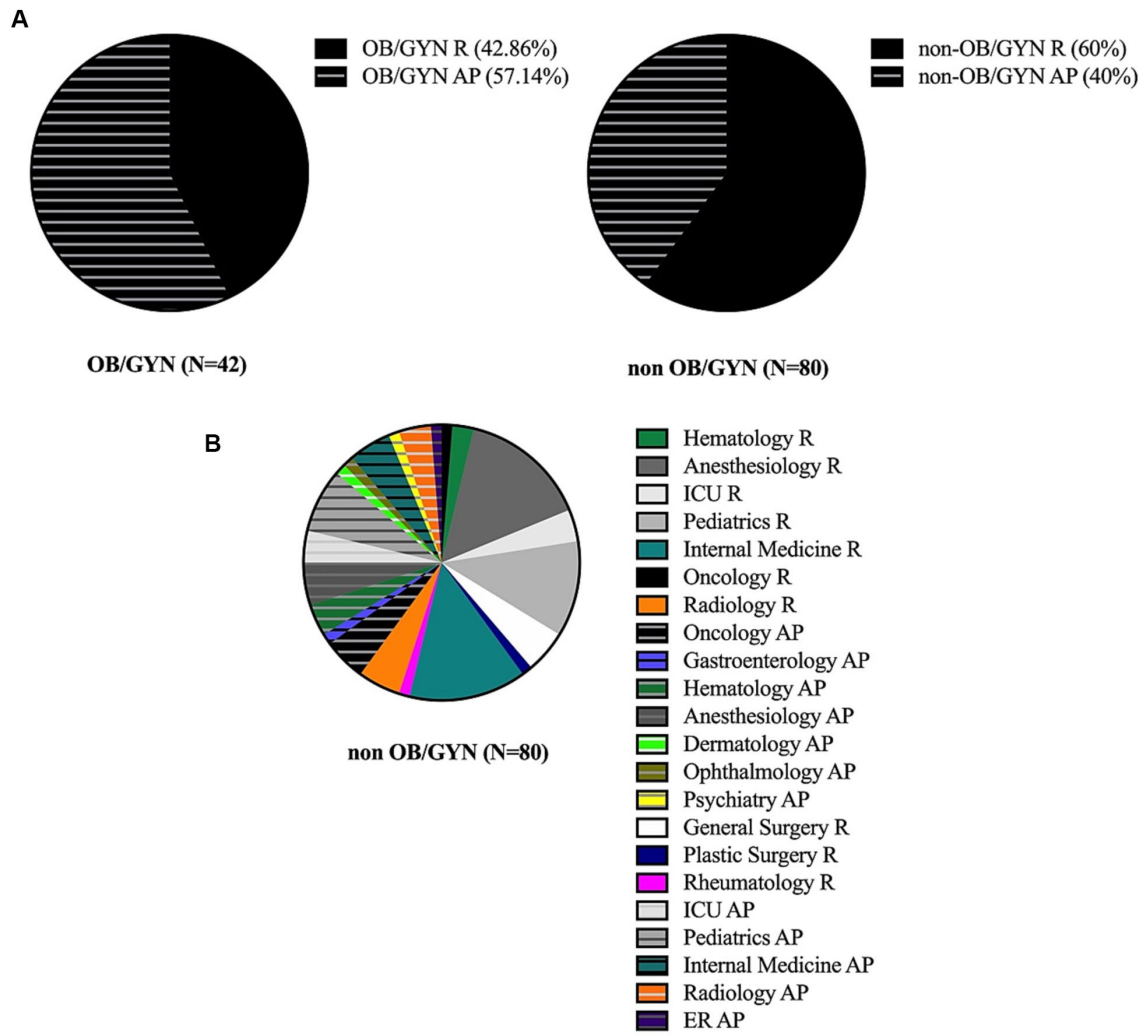
**(A)** Pie chart presentation of seniority for OB/GYN (right panel) and non-OB/GYN (left panel) in the study cohort. R – residents, AP − attending physician. **(B)** Pie chart presentation of specialty distribution of non-OB/GYN in the study cohort. R − residents, AP − attending physician, ICU − intensive care unit, ER − emergency room. Of non-OB/GYN residents: Oncology − 2.08%, Hematology − 4.17%, Anesthesiology − 25%, ICU − 6.25%, Pediatrics − 18.75%, General surgery − 8.33%, Plastic surgery − 2.08%, Internal medicine − 22.93%, Rheumatology − 2.08%, Radiology − 8.33%. Of non-OB/GYN attending physicians: Oncology − 12.5%, Gastroenterology − 3.125%, Hematology − 9.375%, Anesthesiology − 12.5, %, ICU − 9.375%, Pediatrics − 18.75%, Dermatology − 3.125%, Ophthalmology − 3.125%, Internal medicine − 12.5%, Psychiatry − 3.125%. Rheumatology − 9.375%, Radiology − 3.125%.

Out of 45 OB/GYN and 130 non-OB/GYN approached, 42 and 80 agreed to participate, respectively (overall response rate 69.7%, OB/GYN 93.33%, non-OB/GYN 61.53%). Of the 42 OB/GYN physicians who completed the questionnaire, there were 18 residents and 24 attending physicians. Of the 80 non-OB/GYN physicians, 48 were residents and 32 were attending physicians. The two groups comprised of similar proportions of resident and attending physicians participants (*p* = 0.0865). Professional characteristics of the study cohort are presented in Table 1, distribution of the study cohort according to specialty and seniority is presented in Figure 1.

**Table 1:** Demographic characteristics and professional experience of the study cohort.

	OB-R	OB-AP	OB-A	NOB-R	NOB-AP	NOB-A	OB-A vs. NOB-A (*p* value)	OB-R vs. OB-AP (*p* value)	NOB-R vs. NOB-AP (*p* value)	OB-R vs. NOB-R (*p* value)	OB-AP vs. NOB-AP (*p* value)
Age (yrs., mean ± SD)	34.5 ± 3.45	44.47 ± 7.6	40.09 ± 7.87	33.29 ± 3.11	44.54 ± 7.53	37.76 ± 7.71	0.605	0.0005***	<0.0001****	>0.99	>0.99
Married (*N*, %) Yes No	12 (66.67) 6 (33.33)	21 (87.5) 3 (12.5)	33 (78.57) 9 (21.43)	33 (68.75) 15 (31.25)	26 (81.25) 6 (18.75)	59 (73.75) 21 (26.25)	0.66	0.139	0.3	>0.99	0.717
Smoking (*N*, %) Yes No	2 (11.11) 16 (88.89)	2 (8.33) 22 (91.67)	4 (9.52) 38 (90.48)	7 (14.58) 41 (85.42)	6 (18.75) 26 (81.25)	13 (16.25) 67 (83.75)	0.413	>0.999	0.758	>0.99	0.44
Sexually active (*N*, %) Yes No	18 (100) 0 (0)	22 (91.67) 2 (8.33)	40 (95.24) 2 (4.76)	45 (93.75) 3 (6.25)	27 (84.38) 5 (15.63)	72 (90) 8 (10)	0.49	0.498	0.255	0.556	0.686
Age at first sexual activity (yrs., mean ± SD)	19.44 ± 3.95	19.45 ± 2.75	19.45 ± 3.3	19.06 ± 2.61	18.93 ± 2.33	19.01 ± 2.52	> 0.999	> 0.99	> 0.99	> 0.999	>0.999
Condom use (*N*, %) Yes No	5 (27.78) 13 (72.22)	0 (0) 24 (100)	5 (11.9) 37 (88.1)	18 (37.5) 30 (62.5)	11 (34.38) 21 (65.63)	29 (36.25) 51 (63.75)	0.0053**	0.01*	0.816	0.568	0.0013**
Education (yrs., mean ± SD)	20.11 ± 1.76	19.74 ± 1.63	19.9 ± 1.67	19.2 ± 2.11	19.08 ± 3.91	19.16 ± 2.88	0.329	>0.99	>0.99	0.854	>0.999
Professional experience (yrs., mean ± SD)	2.63 ± 1.62	10.52 ± 7.86	6.93 ± 7.06	2.58 ± 1.67	11.53 ± 8.46	6.43 ± 7.27	>0.999	0.0077**	<0.0001****	>0.999	>0.999
Age at first cervical screening (yrs., mean ± SD) <25 yrs. (*N*, %) >25 yrs. (*N*, %)	24.77 ± 3.46 9 (69.23) 4 (30.77)	26.94 ± 5.12 6 (33) 12 (66.67)	26.03 ± 4.57 15 (48.39) 16 (51.61)	21.83 ± 3.59 31 (83.78) 6 (16.22)	24.51 ± 5.17 17 (62.96%) 10 (37.04%)	22.96 ± 4.56 48 (75) 16 (25)	0.0239* 0.019*	>0.999 0.073	0.40 0.08	0.400 0.42	>0.999 0.07

OB-R – OB/GYN residents. OB-AP – OB/GYN attending physicians. NOB-R – non-OB/GYN residents. NOB-AP – non-OB/GYN attending physicians. OB-A – OB/GYN all. NOB-A – non-OBGYN all. Yrs. – years (of age). SD- Standard deviation. * – *p* < 0.05. ** – p < 0.01. *** – *p* < 0.001. ****p < 0.0001.

There were no significant differences in the demographic characteristics and professional experience between the two groups as presented in Table 1. There were no significant differences in the prevalence of the following risk factors for HPV infection and CC: smoking, sexually activity, and average age at initiation of sexual activity. All the above mentioned remained non-significant when participants were stratified based on seniority, as shown in Table 1. It is noteworthy that condom use was less prevalent in OB/GYN attending physicians compared to OB/GYN residents (0% vs. 27.78%, *p* = 0.01) and compared to non-OB/GYNs attending physicians (0% vs. 34.38%, *p* = 0.0013), despite similar proportions of married and sexually active individuals in these groups (Table 1). The age at the performance of first cervical screening was younger in the non-OB/GYNs group (22.96 ± 4.56 years vs. 26.03 ± 4.57 years, *p* = 0. 023) and significantly less OB/GYNs were in compliance with the recommendations of the Israeli Society of Obstetrics and Gynecology for first cervical screening at the age of 25 (48.38% vs. 75%, *p* = 0.01), probably due to a trend in OB/GYN attending physicians (OB/GYN attending physicians 33% non-OB/GYN attending physicians 62.96%, *p* = 0.07). Of note, this difference cannot be attributed to professional differences since the participants were still during their basic medical education at the time.

Table 2 summarizes the differences in general knowledge regarding CC and cervical screening between the groups. A significantly higher proportion of OB/GYNs correctly answered each of the 6 questions asked (question 1 - *p* = 0.0006 questions 2, 4–6 - *p* < 0.001, question 10 -*p* = 0.0018, Supplementary File S2). Notably, less OB/GYN residents knew about the Israeli Society of Obstetrics and Gynecology’s recommendations for upper age limit for cervical screening (50% vs. 83%, *p* = 0.04) and for upper age limit for vaccination (11% vs. 50%, *p* = 0.01). Even so, when asked whether they would recommend HPV vaccination to their patients, the majority of physicians, both OB/GYNs and non-OB/GYNs and in all levels of seniority, answered positively (OB/GYNs – 93%, non-OB/GYNs 86%, *p* = 0.37). Physicians in the OB/GYNs group also demonstrated greater knowledge regarding risk factors for CC as seen by significantly higher calculated risk factor awareness indices (5.78 vs. 4.48, *p* < 0.001), with no difference between residents and attending physicians (Table 2).

**Table 2:** Knowledge of cervical screening and HPV vaccination among OB/GYN and non – OB/GYN female physicians.

	OB-R	OB-AP	OB-A	NOB-R	NOB-AP	NOB-A	OB-A vs. NOB-A (*p* value)	OB-R vs. OB-AP (*p* value)	NOB-R vs. NOB-AP (*p* value)	OB-R vs. NOB-R (*p* value)	OB-AP vs. NOB-AP (*p* value)
Goal of cervical screening (*N*, %) Right answer Wrong answer	18 (100) 0 (0)	24 (100) 0 (0)	42 (100) 0 (0)	38 (79) 10 (21)	24 (77) 7 (23)	62 (77) 17 (23)	0.0006***	>0.99	>0.99	0.051	0.014*
Recommended screening frequency (*N*, %) Right answer Wrong answer	16 (89) 2 (11)	21 (87) 3 (13)	37 (88) 5 (12)	18 (38) 30 (62)	10 (32) 21 (68)	28 (35) 51 (65)	< 0.0001****	> 0.99	0.81	0.0002***	< 0.0001****
Recommended age first cervical screening (*N*, %) Right answer Wrong answer	11 (61) 7 (39)	19 (79) 5 (21)	30 (71) 12 (29)	6 (12) 42 (88)	4 (13) 27 (87)	10 (12) 69 (88)	< 0.0001****	0.302	> 0.99	0.0002***	< 0.0001****
Recommended age last cervical screening (*N*, %) Right answer Wrong answer	9 (50) 9 (50)	20 (83) 4 (17)	29 (69) 13 (31)	5 (10) 43 (90)	3 (10) 28 (90)	8 (10) 71 (90)	< 0.0001****	0.04*	> 0.99	0.0012**	< 0.0001****
Aware of vaccine type (*N*, %) Right answer Wrong answer	12 (67) 6 (33)	17 (71) 7 (29)	29 (69) 13 (31)	13 (27) 35 (73)	9 (29) 22 (71)	22 (28) 57 (72)	< 0.0001****	> 0.99	> 0.99	0.0046**	0.0029**
Upper age of vaccine (*N*, %) Right answer Wrong answer	2 (11) 16 (89)	12 (50) 12 (50)	14 (33) 28 (67)	3 (6) 45 (94)	4 (13) 27 (87)	7 (9) 72 (91)	0.0018**	0.01*	0.423	0.608	0.006**
Recommends vaccination (*N*, %) 1 Yes 2 No	16 (89) 2 (11)	23 (96) 1 (4)	39 (93) 3 (7)	44 (92) 4 (8)	24 (77) 7 (23)	68 (86) 11 (14)	0.374	0.567	0.099	0.66	0.119
Risk factor awareness index (out of 7 ± SD)	5.55 ± 1.15	5.95 ± 0.8	5.78 ± 0.97	4.43 ± 1.08	4.56 ± 0.95	4.48 ± 1.03	< 0.0001****	> 0.99	> 0.99	0.006**	< 0.0001****

OB-R – OB/GYN residents. OB-AP – OB/GYN attending physicians. NOB-R – non-OB/GYN residents. NOB-AP – non-OB/GYN attending physicians. OB-A – OB/GYN all. NOB-A – non-OBGYN all. HPV – Human Papillomavirus. SD – Standard deviation. * – *p* < 0.05. ** – *p* < 0.01. *** – *p* < 0.001. ****p < 0.0001.

Data regarding cervical screening status is presented in Table 3. In average, longer time has passed since the last cervical screening in the OB/GYNs group compared with the non-OBGYNs group (3.305 years vs. 2.26 years, respectively, *p* = 0.0099) but a similar proportion of physicians had performed a cervical screening test in the last 3 years (OB/GYN – 75% non-OB/GYN – 83%, *p* = 0.3). Of note, a higher percentage of residents, both OB/GYNs and non- OBGYNs were vaccinated against HPV compared to their senior counterparts (OB/GYNs −38.89% vs. 4.76%, *p* = 0.0149, non-OB/GYNs 51.06% vs. 15.38%, *p* = 0.0028). Excluded from these calculations were 3 OB/GYN attending physicians, 1 non-OB/GYN resident and 6 non-OB/GYN attending physicians who were older than 45 years at the time HPV vaccination was available in Israel. About half of the unvaccinated physicians agreed to share the reason for their choice. Interestingly, 60% of OB/GYN resident and attending physicians as well as of non-OB/GYN attending physicians did not vaccinate because they had a regular sex partner. The remaining 40% did not vaccinate “because of their age.” Out of the unvaccinated non-OB/GYN attending physicians who revealed their motives, 16.66% justified their choice in having a regular sex partner and 83.34% stated their reason was age-related. Only half of OB/GYNs (residents – 50%, attending physicians – 66.67%, *p* = 0.348) initiated their own cervical screening test, similar to non-OB/GYNs. Among non-OB/GYNs, residents showed a higher initiative toward undergoing cervical screening compared to attending physicians, although not significant (47.92% vs. 37.5%, *p* = 0.49). Overall, OBGYNs tended to initiate the test more than non – OBGYNs although also not significant (59.52% vs. 43.75%, *p = 0.127).*

**Table 3:** Cervical screening and HPV vaccination compliance in OB/GYN and non-OB/GYN female physicians.

	OB-R	OB-AP	OB-A	NOB-R	NOB-AP	NOB-A	OB-A vs. NOB-A (*p* value)	OB-R vs. OB-AP (*p* value)	NOB-R vs. NOB-AP (*p* value)	OB-R vs. NOB-R (*p* value)	OB-AP vs. NOB-AP (*p* value)
Time from last cervical screening (yrs., mean ± SD) <3 years (*N*, %) >3 years (*N*, %)	3.62 ± 3.21 13 (81.25) 3 (18.75)	3.05 ± 1.38 14 (70) 6 (30)	3.30 ± 2.35 27 (75) 9 (25)	2.2073 ± 1.58 35 (85.37) 6 (14.63)	2.35 ± 2.02 22 (81.48) 5 (18.52)	2.26 ± 1.79 57 (83.82) 11 (16.18)	0.0099** 0.3	>0.99 0.7	>0.99 0.742	0.31 0.7	0.208 0.489
Time between last cervical screening and previous one (yrs., mean ± SD)	3.68 ± 3.17	3 ± 1.41	3.30 ± 2.35	2.5 ± 1.32	3.41 ± 4.125	2.904 ± 2.980	0.691	>0.99	>0.99	>0.99	>0.99
Cervical screening initiator Patient (*N*, %) Physician (*N*, %)	9 (50) 9 (50)	16 (66.67) 8 (33.33)	25 (59.52) 17 (40.48)	23 (47.92) 25 (52.08)	12 (37.5) 20 (62.5)	35 (43.75) 45 (56.25)	0.127	0.348	0.49	>0.99	0.057
HPV vaccination status ^ Positive (*N*, %) Negative (*N*, %)	7 (38.89) 11 (61.11)	1 (4.76) 20 (95.24)	8 (20.51) 31 (79.49)	24 (51.06) 23 (48.94)	4 (15.38) 22 (84.62)	28 (38.36) 45 (61.64)	0.059	0.0149*	0.0028**	0.418	0.362

^Excluding cases over vaccination age limit.

OB-R – OB/GYN residents. OB-S – OB/GYN attending physicians. NOB-R – non-OB/GYN residents. NOB-AP – non-OB/GYN attending physicians. OB-A – OB/GYN all. NOB-A – non-OBGYN all. HPV – Human Papillomavirus. yrs. – years (of age). SD – Standard deviation. *p < 0.05. **p < 0.01. ***p < 0.001. ****p < 0.0001.

## Discussion

One hundred and twenty-two female physicians (42 OB/GYNs, 80 non-OB/GYNs) accepted our request to complete a two-part questionnaire regarding their knowledge on CC and cervical screening as well as their compliance with the test. Our results showed that OB/GYNs had greater knowledge about CC and the importance of cervical screening tests (as expected) compared to non-OB/GYNs. For the most part, residents and attending physicians in the two groups were equally educated to the extent covered in the study questionnaire. Less OB/GYN residents knew about the Israeli Society of Obstetrics and Gynecology’s recommendations for upper age limit for cervical screening and about the upper age limit for vaccination compares to OB/GYN attending physicians. During residency, the residents do not work outside the hospital. Since routine gynecological examinations, including cervical screening, are carried out by the principal OB/GYNs in their clinics, the residents are rarely exposed to them. They acquire the theoretical knowledge as part of their residency training but are required to use it seldomly and are thus less familiar with recommendations’ subtleties. We are currently implementing a periodic briefing of cervical screening and HPV vaccination guidelines into the resident’s curriculum to increase awareness and knowledge from the earliest stages of residency. Despite their broader knowledge, OB/GYNs did not initiate CC screening more than non-OB/GYNs nor did they comply more with the recommended timeframe for cervical screening (once every 3 years). Regardless of their knowledge, all four compared groups highly recommended HPV vaccine. Surprisingly, significantly more residents were vaccinated against HPV compared to their senior counterparts. Somewhat similar results were recently published by Soylar et al., who conducted a study evaluating the knowledge, attitude, and practice regarding cancer screening tests among health workers in a university hospital in Turkey. The authors reported that although 50.3% of the health workers demonstrated correct knowledge of the timing of Pap smear and 94.85% demonstrated positive attitude toward the test, only 4.2% of them practiced it. Contrary to our results, Soylar et al. found that as the age and years of experience of the workers increased, they were more likely to undergo screening tests. Of note, only 17.7% of the study population were physicians and all of them were residents (12).

Additional studies have examined the compliance of medical staff to Pap testing. Kabacaoglu et al. questioned 524 female health personnel (18.3% physicians and 81.7% nurses) regarding breast and CC prevention practice and showed that while 26.3% of participants thought about taking an HPV test, only 4.4% did so. 19.5% of married participants had a Pap smear conducted regularly. The most important causes for not performing tests for early diagnosis of breast and cervical cancers were “forget and neglect” (13). Can et al. examined the awareness of 327 female health employees (midwives 43.7%, nurses 40.4%, physicians 6.4%, emergency medical technicians and others 9.5%) working in primary health care services to CC and its risk factors. They reported that a large proportion of the subjects had little knowledge regarding CC and inadequate screening practice (14). Another study conducted by Oranratanaphan included 78 participants (31.8% nurses, 43.9% nurse aids and 24.2% “others”) and showed that while their knowledge about the importance of the Pap smear, early detection and the treatment of early-stage CC was adequate, the participants’ awareness of CC risk factors was relatively low. The main reasons for avoiding Pap smear screening were fear of vaginal examination (27.6%), embarrassment (26.3%), lack of any symptoms (22.4%) and being busy (17%) (15).

Practicing medicine is a highly stressful job. Stress has been shown to be associated with less practice of health promoting behaviors. Tucker et al. surveyed 2,247 staff nurses working in a large medical center in the US and showed that perceived stress levels were negatively associated with overall health-promoting behavior indices (16). Furthermore, in 2016, a cross-sectional study of over 30,000 female nurses working in 100 hospitals across Taiwan demonstrated that nurses were less likely to have Pap smear screening that the general population. Amazingly, each point increase in stress index decreased the likelihood of Pap smears (17).

Doctors are notoriously known for being the worst patients. Haran et al. reported extremely low compliance of physicians and nurses in administrating iron supplements for their own children. Nurses were significantly more compliant compared to physicians. The degree of seniority also affected compliance - specialists were significantly less compliant compared to interns and certified nurses were also less compliant compared to uncertified nurses (18). This was also the case for HPV vaccination in our study group but not for cervical screening initiative. Including only physicians who were eligible for vaccination, the proportion of OB/GYNs who were vaccinated did not differ significantly from non-OB/GYNs (20.51% vs. 38.36%, *p* = 0.059). Only 1 OB/GYN attending physician was vaccinated out of 24 OB/GYN attending physicians who were eligible. 90% of unvaccinated OB/GYN attending physicians revealed their rationale for not vaccinating (60% - “regular sexual partner” 40% - “age”) implying that even though they are trained to provide evidence-based care to their patients, they might still hold onto personal beliefs or practices that align with their professional biases. Since attending OB/GYNs practice different subspecialties (obstetrics, fertility, general gynecology, gynecologic oncology), keeping up with the latest research, guidelines, and recommendations outside their day-to-day subspecialty can be overwhelming. This information overload can lead to a lack of time or motivation to critically evaluate the evidence for every personal health decision. In addition to all the above-mentioned influencers, physicians are not immune to the well-documented phenomenon wherein individuals resist behavior changes or interventions that are meant to improve their own health. OB/GYNs might rationalize that their medical expertise exempts them from needing to adhere to certain evidence-based recommendations.

### Strengths and limitations

Our study has several strengths. First, the study was performed in a large medical center in the Tel-Aviv metropolitan area, which limited the number of participants but enabled a homogenous background of the participants. Second, the questionnaires were anonymous, which assured our subjects’ privacy and helped them answer the questions honestly and raised our study’s reliability.

Our study has two major limitations. First, participants were included on voluntary basis, potentially subjecting our results to volunteer bias. It is well accepted that volunteers might be less likely to put themselves forward for studies of behaviors that are less socially acceptable and are related to the studied condition or disease (19). Ensuring anonymization in both delivery and answering of the questioner was implemented to minimize that effect. Our study results are in line with previous, less focused, studies, strengthening the notion that this bias was probably not significant. Second, our participants were inquired about past events, subjecting their answers to recall bias (20). We believe that physicians are specifically attentive to their medical history thus decreasing the chances of recall bias effecting our study results. If any of the biases did affect out study to some extent, both OB/GYNs and non-OBGYNs were probably equally affected.

## Conclusion

Together with our results, it appears that medical knowledge does not necessarily correlate with adequate personal practice when it comes to doctor’s personal health promoting behaviors. Our study showed the same trend by demonstrating equal compliance of OB/GYNs with recommended screening test intervals, self-initiation of cervical screening and HPV vaccination as non-OB/GYN when intuitively, they are expected to perform better. Physicians in their residency training are probably more updated in other fields of medicine compared to attending physicians, resulting in better general personal health habits out of their field of specialty.

To our knowledge, this is the first study to examine if OB/GYNs “practice what they preach.” We showed that female OB/GYN’s knowledge of the importance of cervical screening and their accessibility to cervical screening services, do not improve their compliance for test performance or HPV vaccination. We have learned from former and the current study that physician’s personal health is also influenced by their workload and schedule, which was further highlightened during COVID19. As healthcare systems around the world faced immense strain during the pandemic that led to delays in routine care and a shift in priorities toward pandemic-related care, not only patients but also healthcare workers themselves were affected. Physicians might have postponed or neglected important preventive care and screenings, creating a backlog of unaddressed health needs that could have lasting consequences. An effort should be made by OB/GYNs and their professional organizations to adhere to cervical screening guidelines and HPV vaccination and set an example for the general population. This could be achieved by creating a framework of annual check-ups, preferably as single-day sessions including several physician encounters and screening for common health problems.

## Data availability statement

The original contributions presented in the study are included in the article/Supplementary material, further inquiries can be directed to the corresponding author/s.

## Ethics statement

The study was conducted according to the guidelines of the Declaration of Helsinki, and approved by the Institutional Review Board of Tel Aviv Sourasky Medical Center (0796–17 – TLV, approved Feb 2018). The studies were conducted in accordance with the local legislation and institutional requirements. Written informed consent for participation was not required from the participants or the participants’ legal guardians/next of kin due to study design - Completing the questionnaire expresses informed consent.

## Author contributions

GH: Conceptualization, Investigation, Methodology, Writing – original draft, Data–curation, Project–administration, Visualization. YO: Conceptualization, Data–curation, Investigation, Methodology, Writing – original draft, Project administration, Visualization. NM: Formal–analysis, Methodology, Writing–review–&–editing. EF: Writing–review–&–editing, Data–curation, Investigation, Resources. DG: Writing–review–&–editing, Methodology, Supervision, Validation. YR: Methodology, Supervision, Validation, Writing–review–&–editing, Conceptualization, Data–curation, Formal–analysis, Investigation, Project administration, Visualization, Writing – original draft.

## Funding

The author(s) declare that no financial support was received for the research, authorship, and/or publication of this article.

## Conflict of interest

The authors declare that the research was conducted in the absence of any commercial or financial relationships that could be construed as a potential conflict of interest.

## Publisher’s note

All claims expressed in this article are solely those of the authors and do not necessarily represent those of their affiliated organizations, or those of the publisher, the editors and the reviewers. Any product that may be evaluated in this article, or claim that may be made by its manufacturer, is not guaranteed or endorsed by the publisher.
