# Supplementary material for: Knowledge is power? Cervical cancer prevention in female OB/GYNs compared to other female physicians
Source: Front Public Health. 2023 Sep 15;11:1269393. doi: 10.3389/fpubh.2023.1269393 (PMC10540616; doi:10.3389/fpubh.2023.1269393)
Supplement: Supplementary file 1 [file Data_Sheet_1.PDF]

Supplementary file 1. Questionnaire - Part 1/2- Personal data.

1- Status:
   - o Resident
   - o Attending physician

2- Department _____________

3- Education_______________

4- Seniority________________

5- Age____________________

6- Marital status
   - o Married
   - o Single
   - o Divorced
   - o Widow

7- Smoking
   - o Yes (for how many years? ______, How many cigarettes per day? ________)
   - o No

8- Past surgeries____________

9- Past medical history_______

10- Regular menses
   - o Yes
   - o No

11- Sexually active
   - o Yes

- No

12- Age at first intercourse_____

13- Number of sexual partners____

14- Sexual orientation

- Heterosexual
- Homosexual
- Bisexual
- Other

15- Type of contraceptive method in use

- Oral contraceptive.
- Intrauterine device.
- Condom
- Other
- None

16- Condom use

- Yes
- No

17- Post coital bleeding

- Yes
- No

*For questions 18-22, “cervical screening” includes performances of Pap or HPV testing.

18- Age at first cervical screening test performance _______________

19- When was your last cervical screening test preformed? ________

20- Time period between last two cervical screening tests preformed______

21- Cervical screening test initiator

- Self
- Primary care physician
- OB/GYN
- A friend

22- Have you ever had an abnormal cervical screening test result?

- Yes
- No

23- If yes, what was the pathology/HPV result? ______

24- Have you ever undergone surgical procedures of the uterine cervix?

- Biopsy
- Ablation
- Conization
- None of the above

25- Were you vaccinated against HPV?

- Yes
- No

26- Type of vaccination__________

27- If you were not vaccinated, why? ___________
