# Supplementary material for: Knowledge is power? Cervical cancer prevention in female OB/GYNs compared to other female physicians
Source: Front Public Health. 2023 Sep 15;11:1269393. doi: 10.3389/fpubh.2023.1269393 (PMC10540616; doi:10.3389/fpubh.2023.1269393)
Supplement: Supplementary file 2 [file Data_Sheet_2.PDF]

Supplementary file 2 - Questionnaire – Part 2/2 – knowledge.

Note: For questions 1-5, “cervical screening” includes performances of Pap or HPV testing.

1- What is the objective of cervical screening tests? ___________

Please base your answers to questions 2 – 9 on the Israeli Society of Obstetrics and Gynecology recommendations.

2- How often should cervical screening tests be repeated? ________

3- Is the recommended frequency enough in your opinion?

   Yes

   No

4- What is the recommended age of first cervical screening test? _________

5- What is the recommended age of last cervical screening test? _________

6- What are the different HPV vaccines? ___________

7- Which HPV types do the vaccines protect from? ________

8- What is the recommended age range for HPV vaccination in Israel?

   ____

9- What are the names of the vaccinations approved for use in Israel? ________

10- What is the upper age limit for administration of the HPV vaccines?

   ____

11- Would you recommend HPV vaccination ? ________

12- What are the risk factors for cervical cancer?

   - Multiple partners
   - Contraceptive pill use

- Intra uterine device use
- Smoking
- Family history of cervical cancer
- Circumcision
- Genetic mutation
